# Supplementary material for: Cell type specific transcriptomic differences in depression show similar patterns between males and females but implicate distinct cell types and genes
Source: Nat Commun. 2023 May 22;14:2912. doi: 10.1038/s41467-023-38530-5 (PMC10203145; doi:10.1038/s41467-023-38530-5)
Supplement: Supplementary file 1 — Supplementary Information [file 41467_2023_38530_MOESM1_ESM.pdf]

Supplementary Information

Supplementary Figures

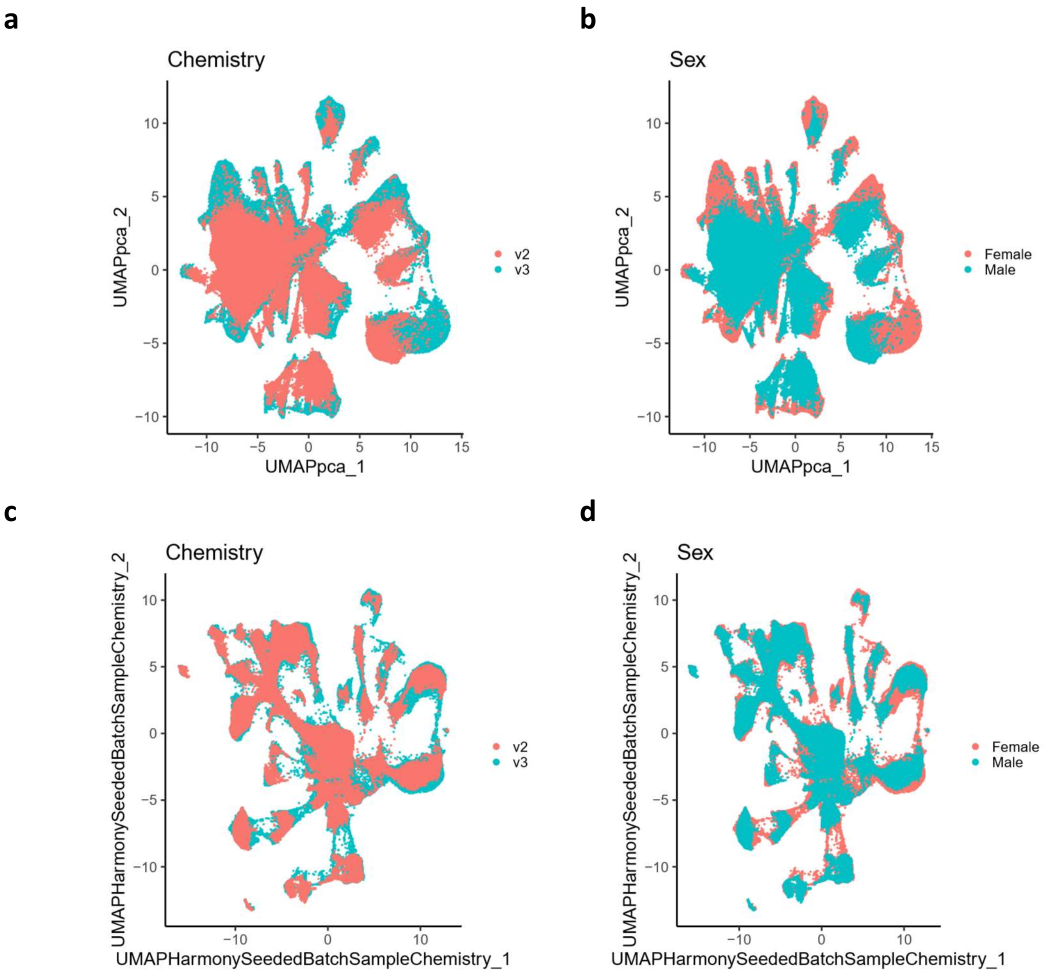

Supplementary Figure 1: UMAP plots using uncorrected and Harmony corrected PCA components. a-b) UMAP plot using uncorrected PCA components colored by chemistry and sex. c-d) UMAP plot using Harmony corrected PCA components colored by chemistry and sex. For UMAPs we used all 100 PC components or Harmony corrected PC components with other parameters set to default. Source data are provided as a Source Data file.

a

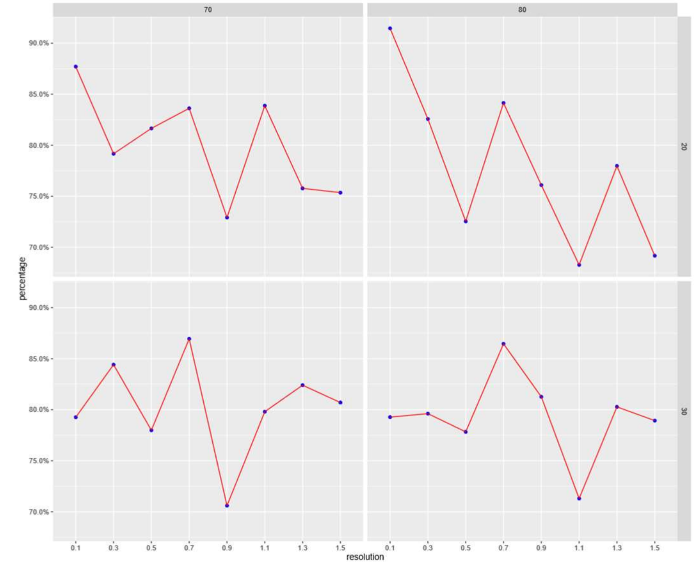

b

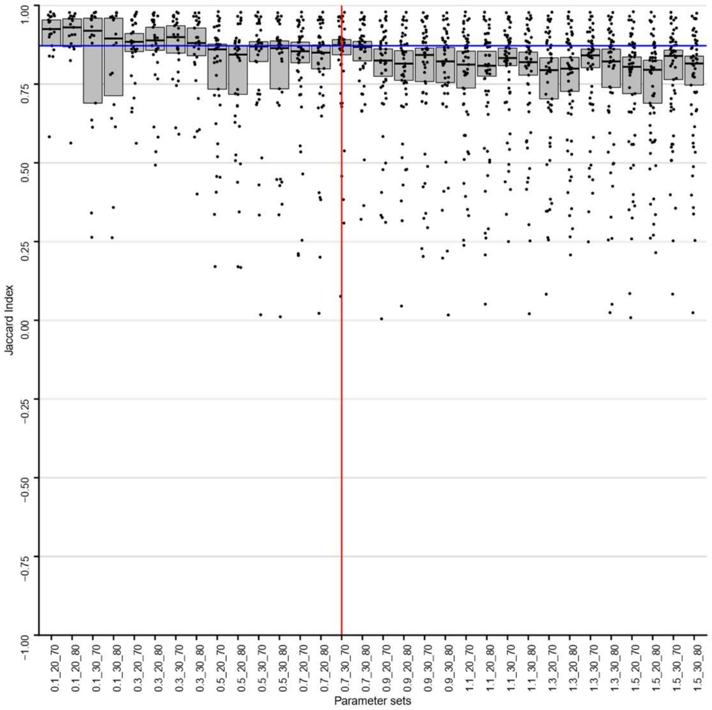

Supplementary Figure 2: Assessment of clustering parameters. a) scclusteval output showing the percentage of nuclei in stable clusters, as assessed by sub-sampling and Jaccard index calculation, using a range of clustering parameters. b) Boxplots showing the median Jaccard index for each cluster across sub-sampling with different parameter combinations for clustering. The

individual dots correspond to the median Jaccard index, across all sub-samplings, for each cluster obtained using the given set of parameters. The boxes represent the bootstrapped 95% confidence interval for the median of the median Jaccard index across all clusters, for the given set of clustering parameters. The box starts at the lower bound of the confidence interval and extends to the higher bound, while the line in the center represents the actual median. The clustering parameters that provide the highest number of clusters, while crossing a bootstrapped threshold of median Jaccard index across clusters, are highlighted. Source data are provided as a Source Data file.

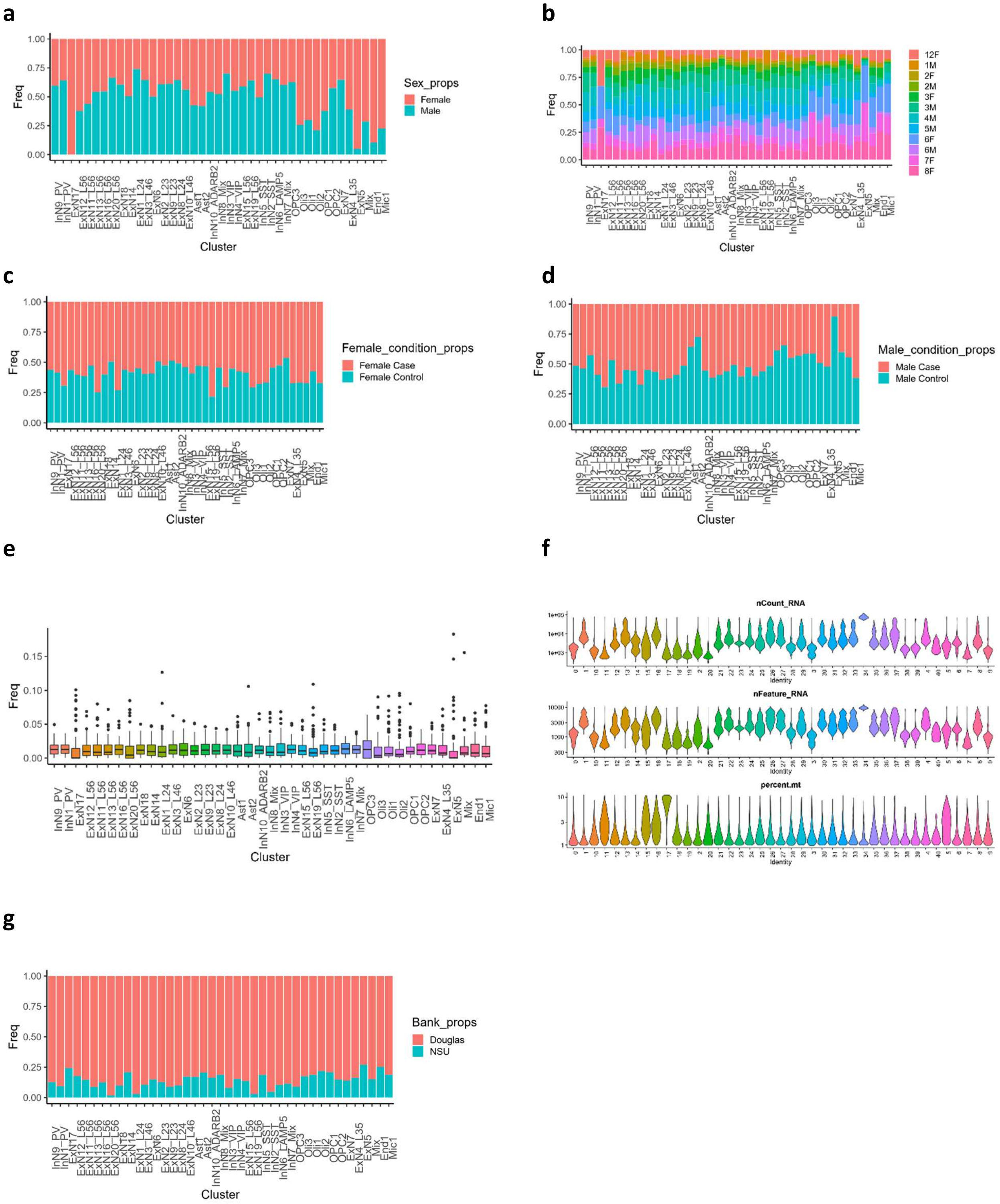
a
Freq
Cluster
Sex_props
Female
Male
b
Freq
Cluster
12F
1M
2F
2M
3F
3M
4M
5M
6F
6M
7F
8F
c
Freq
Cluster
Female_condition_props
Female Case
Female Control
d
Freq
Cluster
Male_condition_props
Male Case
Male Control
e
Freq
Cluster
f
nCount_RNA
nFeature_RNA
percent.mt
Identity
g
Freq
Cluster
Bank_props
Douglas
NSU

Supplementary Figure 3: Evaluation of clusters based on technical parameters. a) Proportions of nuclei from each sex in each cluster. b) Proportion of nuclei from each batch in each cluster. c) Proportion of nuclei from cases and controls among the female nuclei in each cluster. d) Proportion of nuclei from cases and controls among the male nuclei in each cluster. e) Proportion of nuclei from each library (mostly corresponding to subject) in each cluster (for each cluster n= 72 samples, i.e. libraries, from 71 biologically independent subjects). The middle line is the median. The lower and upper hinges correspond to the 25th and 75th percentiles. Upper and lower whiskers extend from the upper or lower hinges to the largest or smallest value no further than 1.5 times the inter-quartile range from the hinge, where the inter-quartile range is the distance between the first and third quartiles. Points beyond the end of the whiskers are plotted individually. f) Violin plots for number of molecules detected, number of genes detected, and percentage of mitochondrial reads per nuclei split by cluster. Mitochondrial gene counts were removed for downstream analysis, after calculating the mitochondrial read percentage. Y-axis is in log-scale. g) Proportions of nuclei from each brain bank in each cluster. For 3a-e, and g, “Freq” stands for frequency representing the proportion of cells. Correspondence between numbered clusters and cluster names in 3f: 0 - ExN1_L24, 1 - ExN2_L23, 2 - Ast1, 3 - Oli1, 4 - ExN3_L46, 5 - ExN4_L35, 6 - Oli2, 7 - Oli3, 8 - InN1_PV, 9 - InN2_SST, 10 - InN3_VIP, 11 - ExN5, 12 - InN4_VIP, 13 - ExN6, 14 - OPC1, 15 - End1, 16 - ExN7, 17 - Mix, 18 - Mic1, 19 - OPC2, 20 - Ast2, 21 - InN5_SST, 22 - ExN8_L24, 23 - ExN9_L23, 24 - ExN10_L46, 25 - InN6_LAMP5, 26 - ExN11_L56, 27 - ExN12_L56, 28 - ExN13_L56, 29 - InN7_Mix, 30 - ExN14, 31 - InN8_ADARB2, 32 - ExN15_L56, 33 - ExN16_L56, 34 - ExN17, 35 - InN9_PV, 36 - InN10_ADARB2, 37 - ExN18, 38 - ExN19_L56, 39 - ExN20_L56, 40 - OPC3. Source data are provided as a Source Data file.

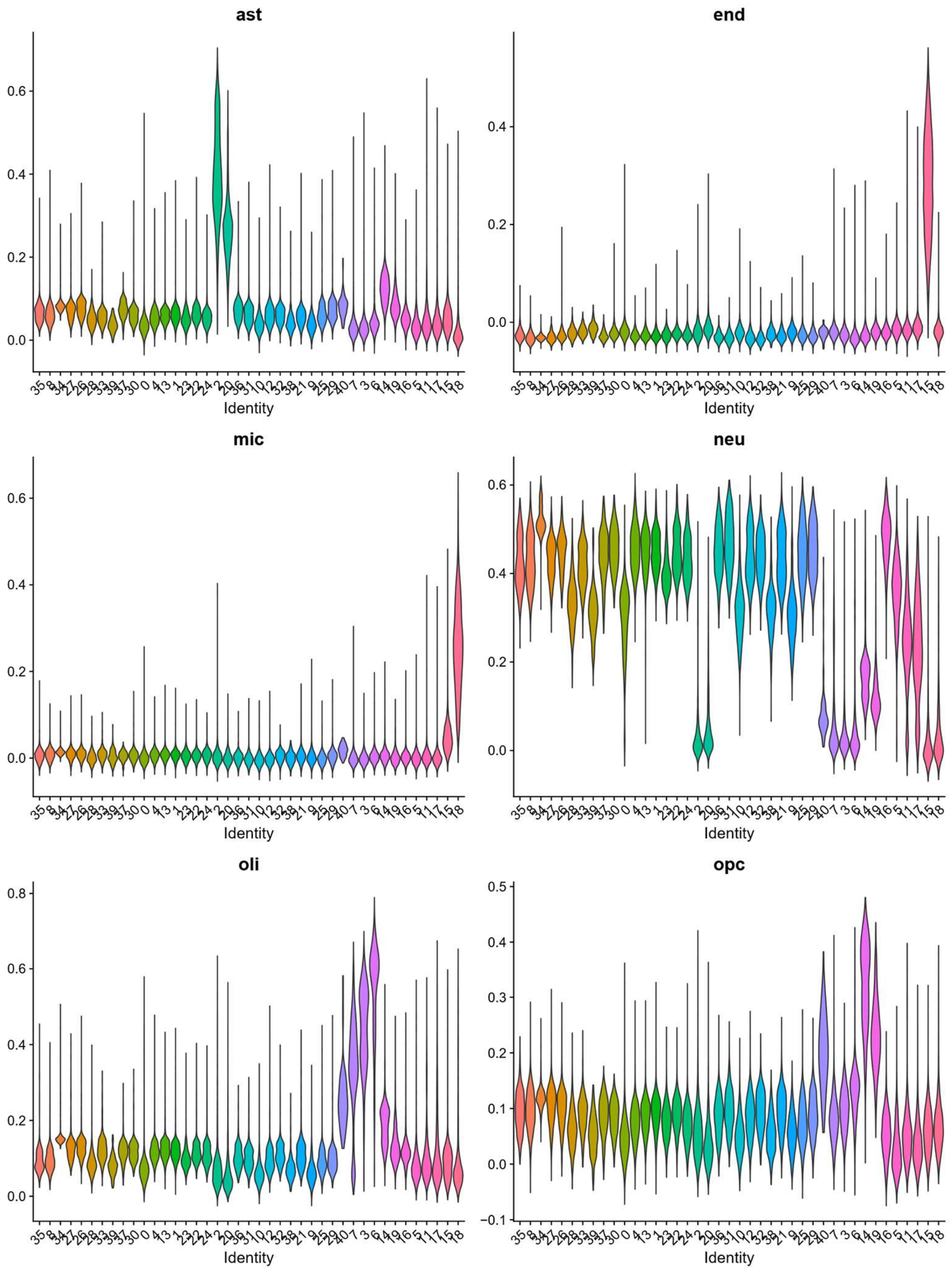

Supplementary Figure 4: Assessment of cell type scores per cluster. Violin plots showing module scores for major brain cell type marker genes from BRETIGEA in each cluster. Correspondence between numbered clusters and cluster names same as in Supplementary Figure 3. Source data are provided as a Source Data file.

a

b

c

d

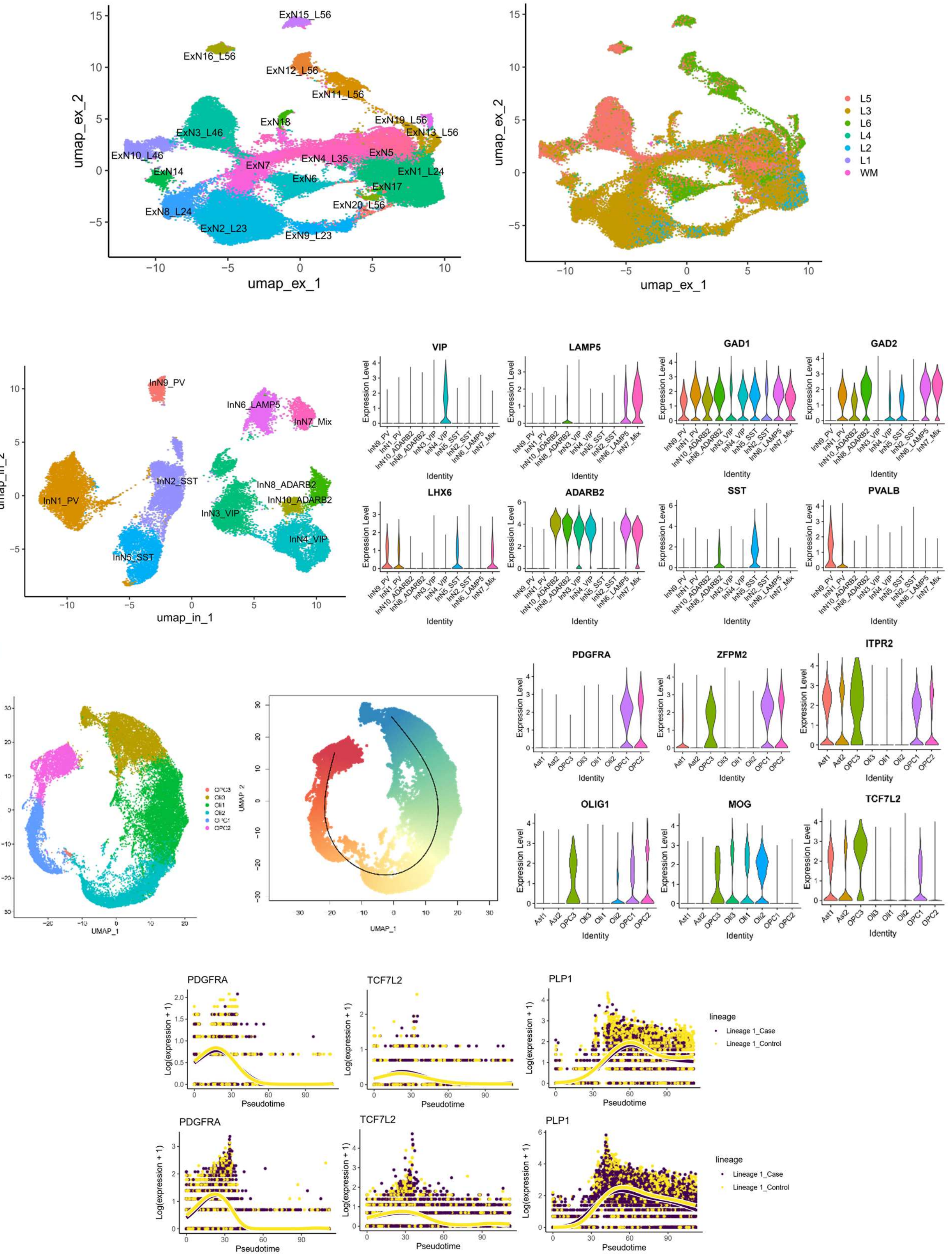
umap_ex_1
umap_ex_2
ExN15_L56
ExN16_L56
ExN12_L56
ExN11_L56
ExN19_L56
ExN13_L56
ExN18
ExN3_L46
ExN10_L46
ExN14
ExN7
ExN4_L35
ExN5
ExN1_L24
ExN6
ExN17
ExN20_L56
ExN8_L24
ExN2_L23
ExN9_L23
L5
L3
L6
L4
L2
L1
WM
umap_in_1
umap_in_2
InN9_PV
InN6_LAMP5
InN7_Mix
InN2_SST
InN8_ADARB2
InN10_ADARB2
InN1_PV
InN3_VIP
InN4_VIP
InN5_SST
VIP
LAMP5
GAD1
GAD2
LHX6
ADARB2
SST
PVALB
Expression Level
Identity
UMAP_1
UMAP_2
PDGFRA
ZFPM2
ITPR2
OLIG1
MOG
TCF7L2
Ast1
Ast2
OPC3
Oli3
Oli1
Oli2
OPC1
OPC2
PLP1
Log(expression + 1)
Pseudotime
lineage
Lineage 1_Case
Lineage 1_Control

Supplementary Figure 5: Annotation of cell sub-types among clusters. a) (left) UMAP plot showing the 20 clusters of excitatory neurons identified. (right) UMAP plot showing the predicted layer labels for excitatory neurons using Seurat label transfer from a spatial transcriptomics dataset of the human dlPFC[1]. b) (left) UMAP plot showing the 10 clusters of inhibitory neurons identified. (right) Violin plots showing the expression of maker genes of inhibitory neurons and their known subtypes. c) (left) UMAP plot showing the 6 oligodendrocyte lineage (OL) clusters. (middle) Same UMAP plot as colored according to the pseudotime trajectory calculated (early to late pseudotime points colored from red to blue). (right) Violin plots showing expression of selected markers of the OL in the respective clusters. d) Smoothed expression fit to depict the variation in expression of selected OL genes along pseudotime using GAMS in males (top) and females (bottom). For UMAPs in (a) and (b) we used the first 50 Harmony components, and n.neighbors = 20. Source data are provided as a Source Data file.

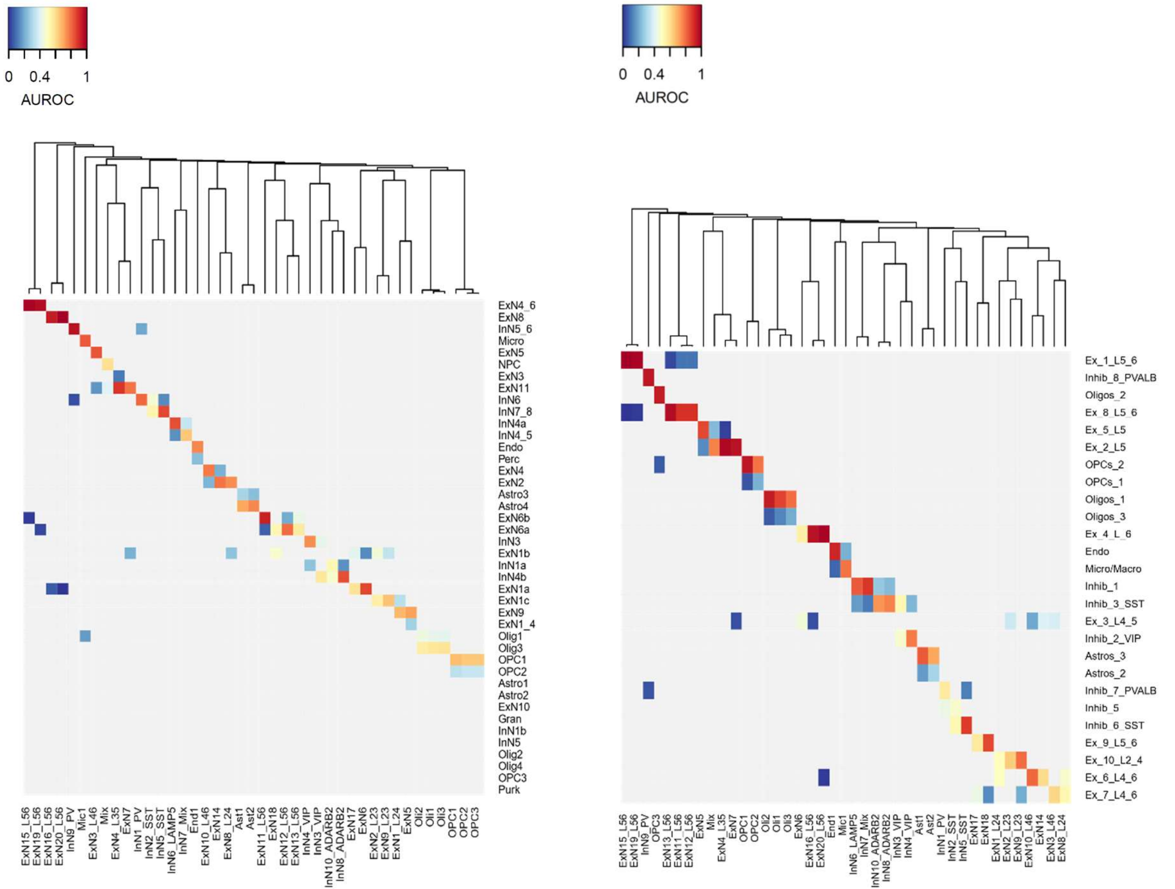

Supplementary Figure 6: Comparison of clustering to published datasets. Best hits plot from MetaNeighbor showing the correspondence between clusters defined in our dataset and clusters defined in (left) the STAB dataset and (right) in our previous analysis of the male cohort alone. Clusters defined in this study are represented on the x-axis while clusters defined in the STAB study and the previous analysis of the male data are represented along the y-axis. Source data are provided as a Source Data file.

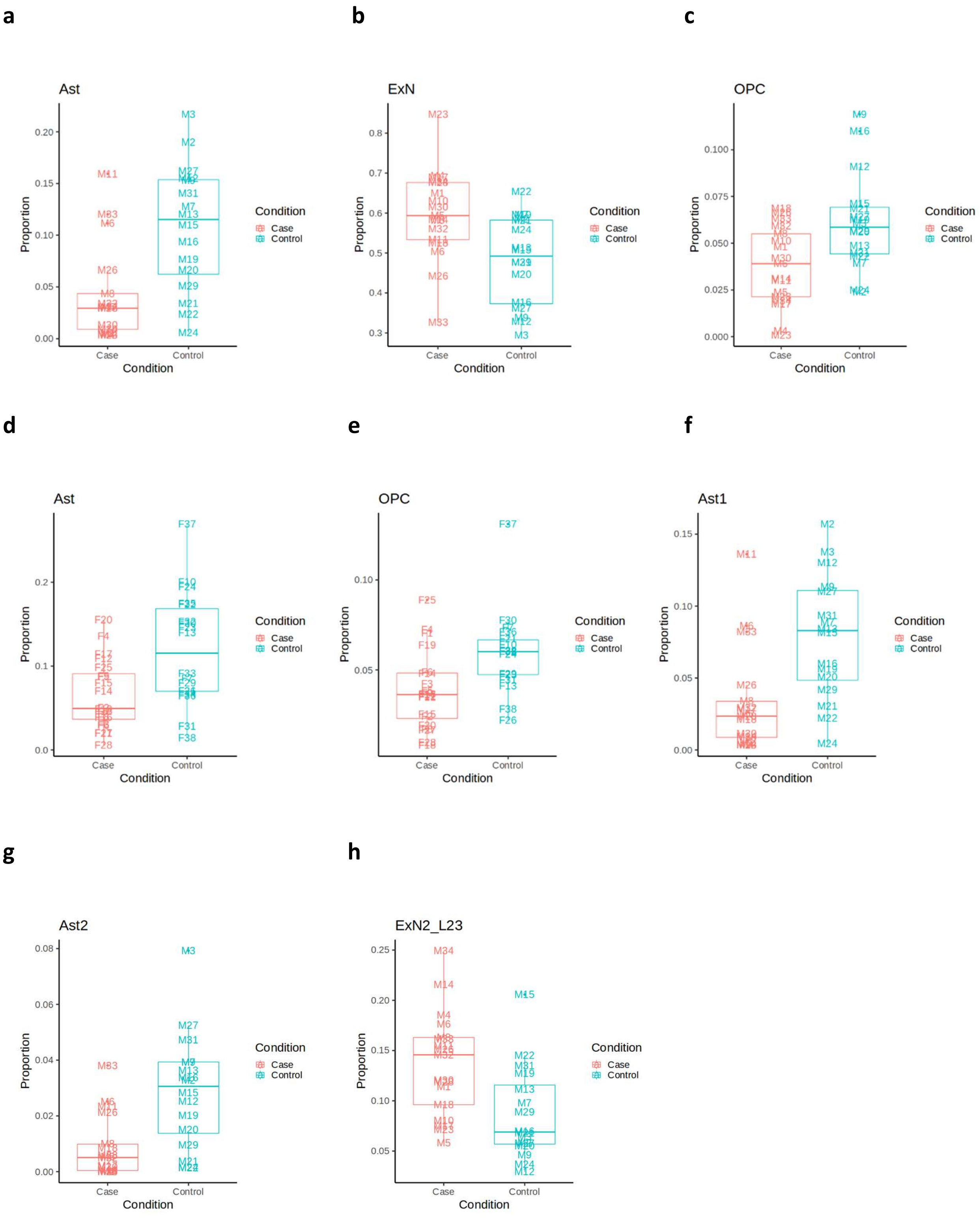
a
b
c
d
e
f
g
h
Ast
ExN
OPC
Ast
OPC
Ast1
Ast2
ExN2_L23
Proportion
Condition
Case
Control

Supplementary Figure 7: Differences in cell type proportion between cases and controls in each sex. Boxplots showing cell type proportions with significant differences between MDD cases and controls, using Wilcoxon tests performed on the male and female datasets separately (n= 17 cases, 16 controls, for males and n=20 cases, 18 controls for females, representing biologically independent samples for each cluster). At the broad level, there was a significant decrease in males in a) Ast (FDR 0.0130) and b) OPC (FDR 0.0325) and an increase in c) ExN (FDR 0.0325). In females, at the broad level the decrease in d) Ast was close to significance (FDR 0.0536) and the decrease in e) OPC (0.0466) was significant. At the cluster level there were no significant differences in the female dataset, but there was a decrease in f) Ast1 (FDR 0.0364) and g) Ast2 (FDR 0.0364) in males and an increase in h) ExN2_L23 (FDR 0.0492). Two-tailed Wilcoxon tests were performed. The middle line is the median. The lower and upper hinges correspond to the 25th and 75th percentiles. Upper and lower whiskers extend from the upper or lower hinges to the largest or smallest value no further than 1.5 times the inter-quartile range from the hinge, where the inter-quartile range is the distance between the first and third quartiles. Points beyond the end of the whiskers are plotted individually. Source data are provided as a Source Data file.

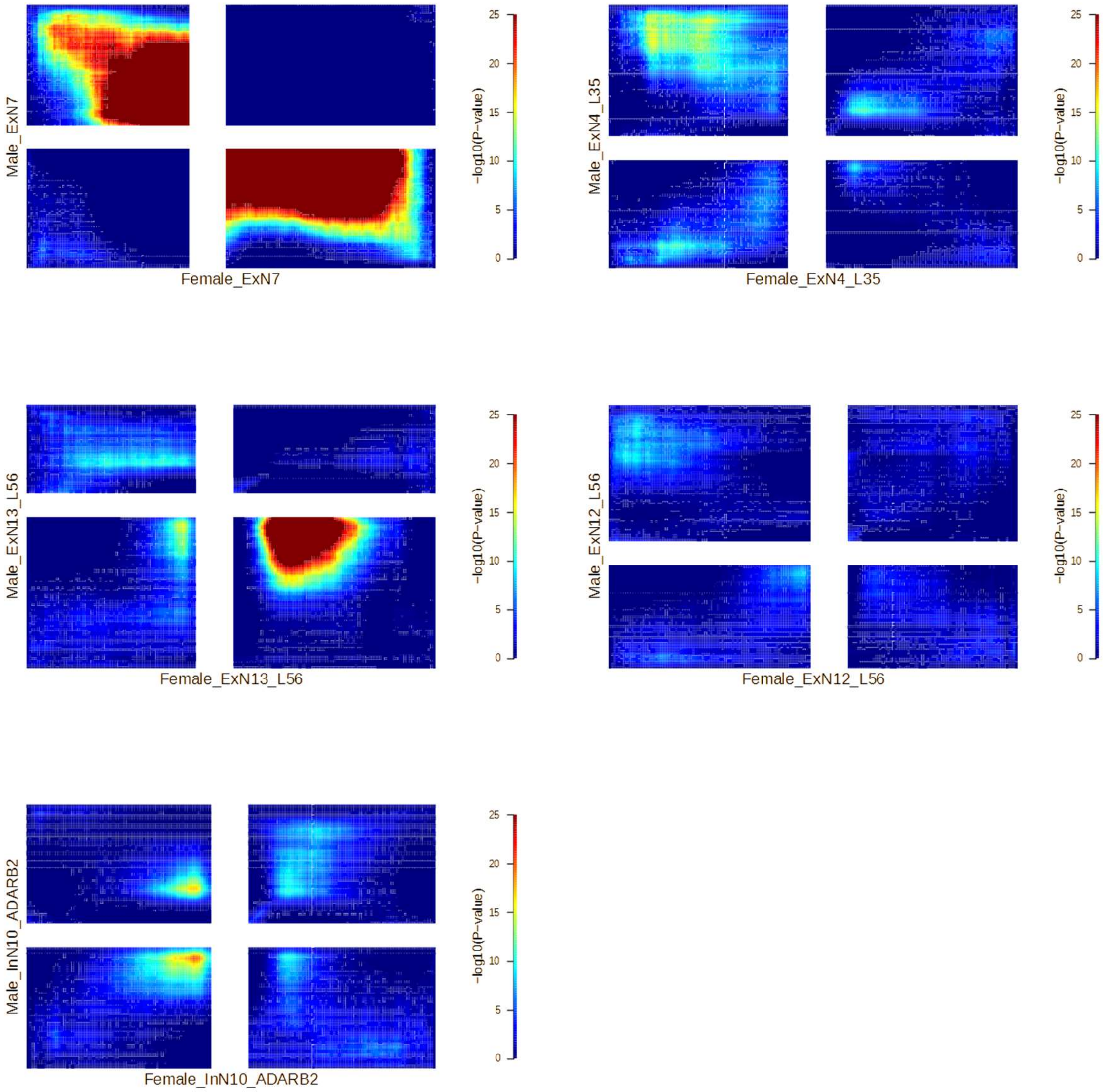

Supplementary Figure 8: RRHO plots for discordant clusters. RRHO plots showing the discordant relationship between males and females for patterns in depression-associated gene expression difference in several excitatory and inhibitory neuronal clusters. Interpretation of the plots is similar to Figure 2. For RRHO plots comparing broad cell types the color scale maximum was set to a -log10(p-value) of 50, and for RRHO plots comparing clusters the color scale maximum was set to a -log10(p-value) of 25 for ease of comparison. RRHO2 uses one-sided hypergeometric tests, the p-values plotted here are uncorrected. Source data are provided as a Source Data file.

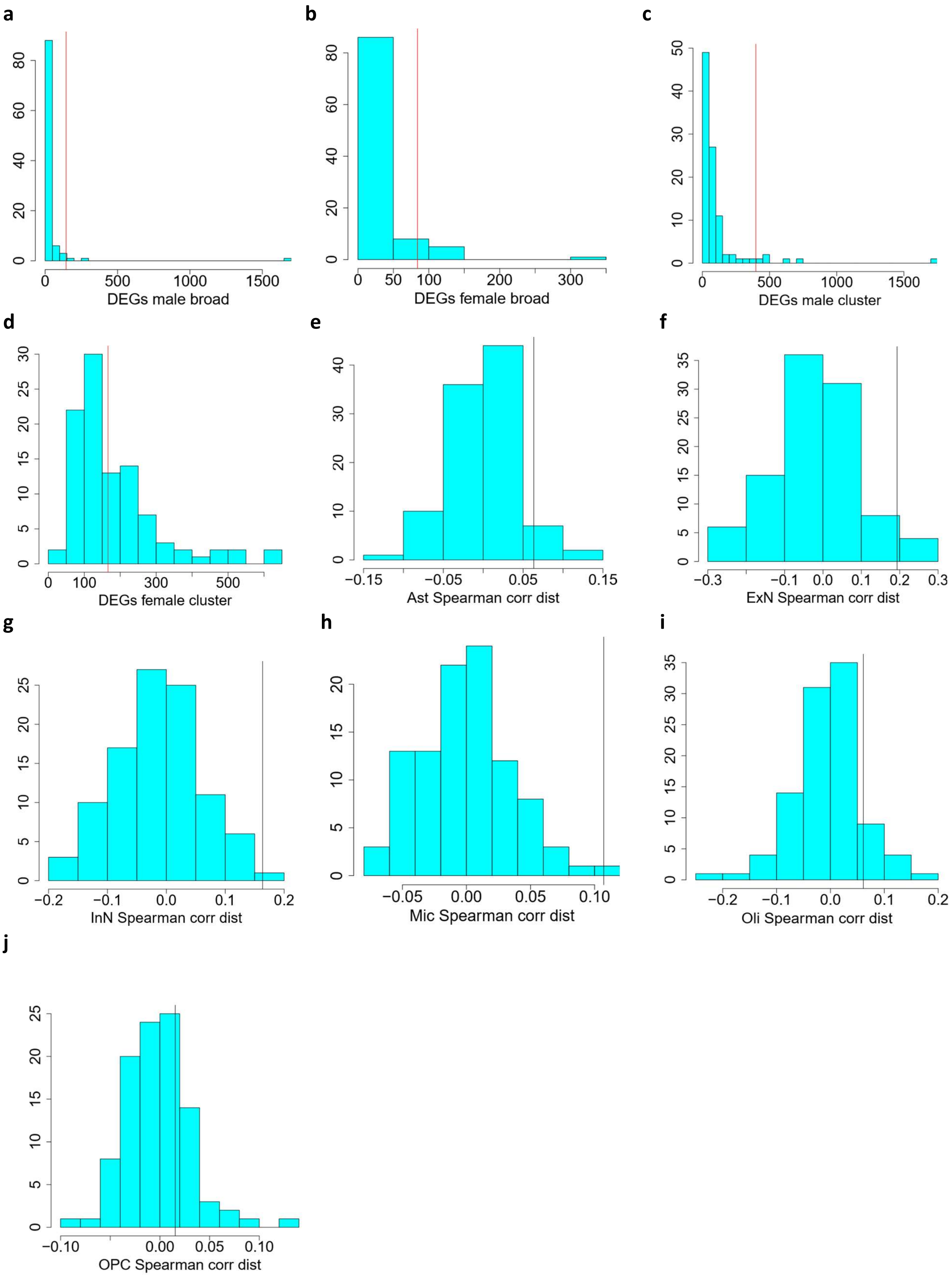
a
0
20
40
60
80
0
500
1000
1500
DEGs male broad
b
0
20
40
60
80
0
50
100
200
300
DEGs female broad
c
0
10
20
30
40
50
0
500
1000
1500
DEGs male cluster
d
0
5
10
15
20
25
30
0
100
300
500
DEGs female cluster
e
0
10
20
30
40
−0.15
−0.05
0.05
0.15
Ast Spearman corr dist
f
0
5
10
15
20
25
30
35
−0.3
−0.1
0.0
0.1
0.2
0.3
ExN Spearman corr dist
g
0
5
10
15
20
25
−0.2
−0.1
0.0
0.1
0.2
InN Spearman corr dist
h
0
5
10
15
20
−0.05
0.00
0.05
0.10
Mic Spearman corr dist
i
0
5
10
15
20
25
30
35
−0.2
−0.1
0.0
0.1
0.2
Oli Spearman corr dist
j
0
5
10
15
20
25
−0.10
0.00
0.05
0.10
OPC Spearman corr dist

Supplementary Figure 9: Permutation assessment for differential expression. Distribution of numbers of unique DEGs using permuted case versus controls labels in: a) males at the broad level, b) females at the broad level, c) males at the cluster level, and d) females at the cluster level. Red vertical lines indicate the number of unique DEGs obtained with real labels and show that the real labels result in higher number of unique DEGs than expected by chance with random case versus control annotations for both sexes at the broad level, and for males at the cluster level. e-j) Distribution of Spearman correlation coefficients when correlating gene scores based on male and female dataset differential gene expression analyses with permuted labels, for broad cell types. The black vertical line denotes the Spearman correlation coefficient obtained with the real case control labels and is higher than the majority of coefficients obtained with randomly permuted cases versus control labels for Ast, ExN, InN, and Mic and to a lesser extent for Oli and OPC. In all plots, the y-axis represents the number of iterations of permutations. Source data are provided as a Source Data file.

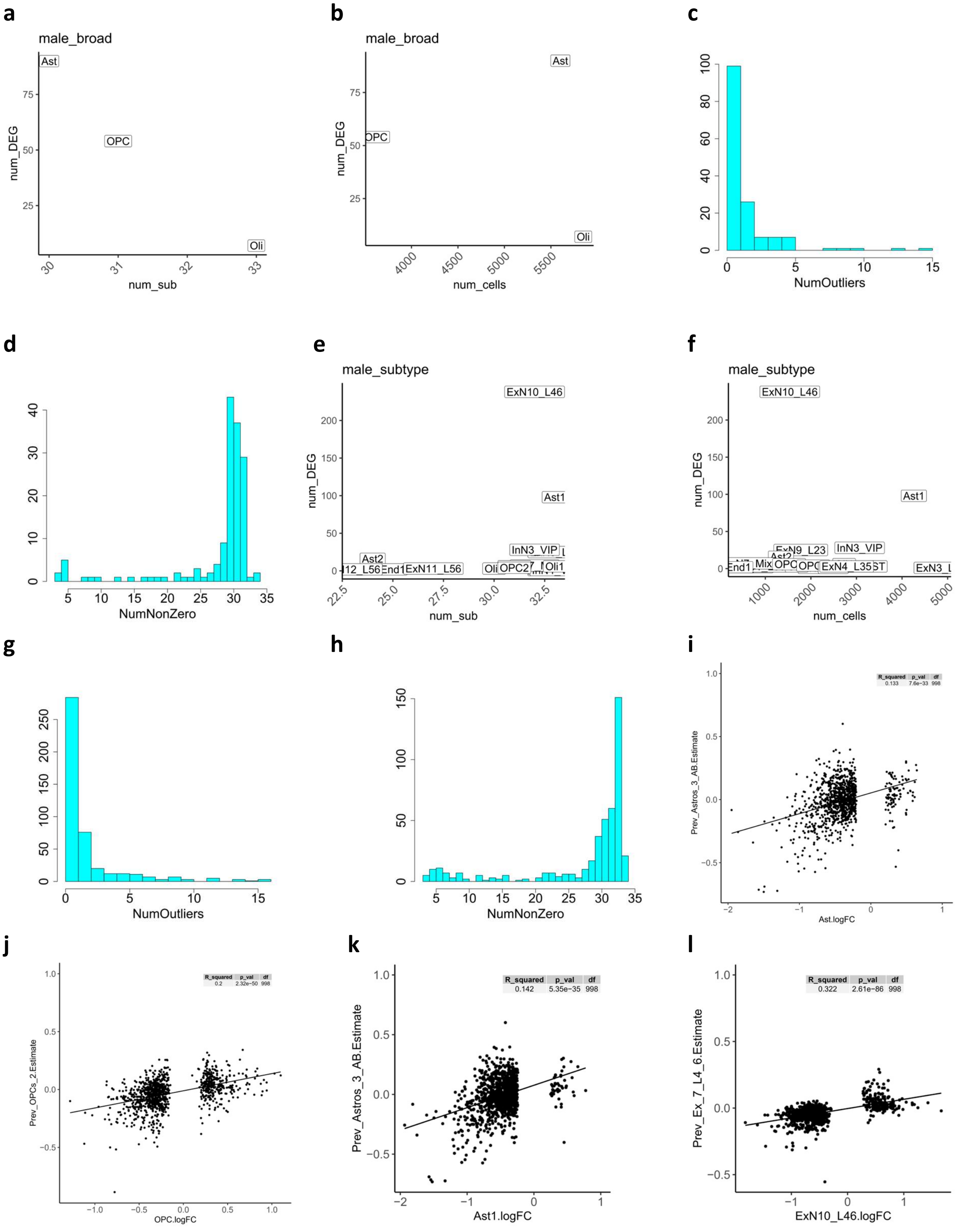
a
male_broad
Ast
OPC
Oli
num_DEG
num_sub
b
male_broad
Ast
OPC
Oli
num_DEG
num_cells
c
NumOutliers
d
NumNonZero
e
male_subtype
ExN10_L46
Ast1
InN3_VIP
Ast2
ExN11_L56
OPC2
Oli1
num_DEG
num_sub
f
male_subtype
ExN10_L46
Ast1
ExN9_L23
InN3_VIP
Ast2
End1
Mix
ExN4_L35
ExN3_L
num_DEG
num_cells
g
NumOutliers
h
NumNonZero
i
Prev_Astros_3_AB.Estimate
Ast.logFC
j
Prev_OPCs_2.Estimate
OPC.logFC
k
R_squared p_val df
0.142 5.35e−35 998
Prev_Astros_3_AB.Estimate
Ast1.logFC
l
R_squared p_val df
0.322 2.61e−86 998
Prev_Ex_7_L4_6.Estimate
ExN10_L46.logFC

Supplementary Figure 10: Evaluation of male differential expression results. Number of DEGs in each broad cell type for males plotted against (a) the number of subjects included in DEG analysis and (b) the total number of nuclei in the broad cell type. For DEGs in males at the broad cell type level (c) the distribution of number of subjects flagged as possible outliers and (d) the distribution of subjects with non-zero expression. Number of DEGs in each cluster for males plotted against (e) the number of subjects included in DEG analysis and (f) the total number of nuclei in the cluster. For DEGs in males at the cluster level (g) the distribution of number of subjects flagged as possible outliers and (h) the distribution of subjects with non-zero expression. Possible outliers were not removed from the analyses but were assessed as a quality metric for DEG analysis. The plots include the Mix cluster results. i-l) Scatter plots showing the relationship between (linear regression and corresponding statistics) the estimated effects per gene from our previous analysis of the male data and the log fold changes from our current analysis for cell type specific case-control differences in males for similar pairs of clusters. Source data are provided as a Source Data file.

a

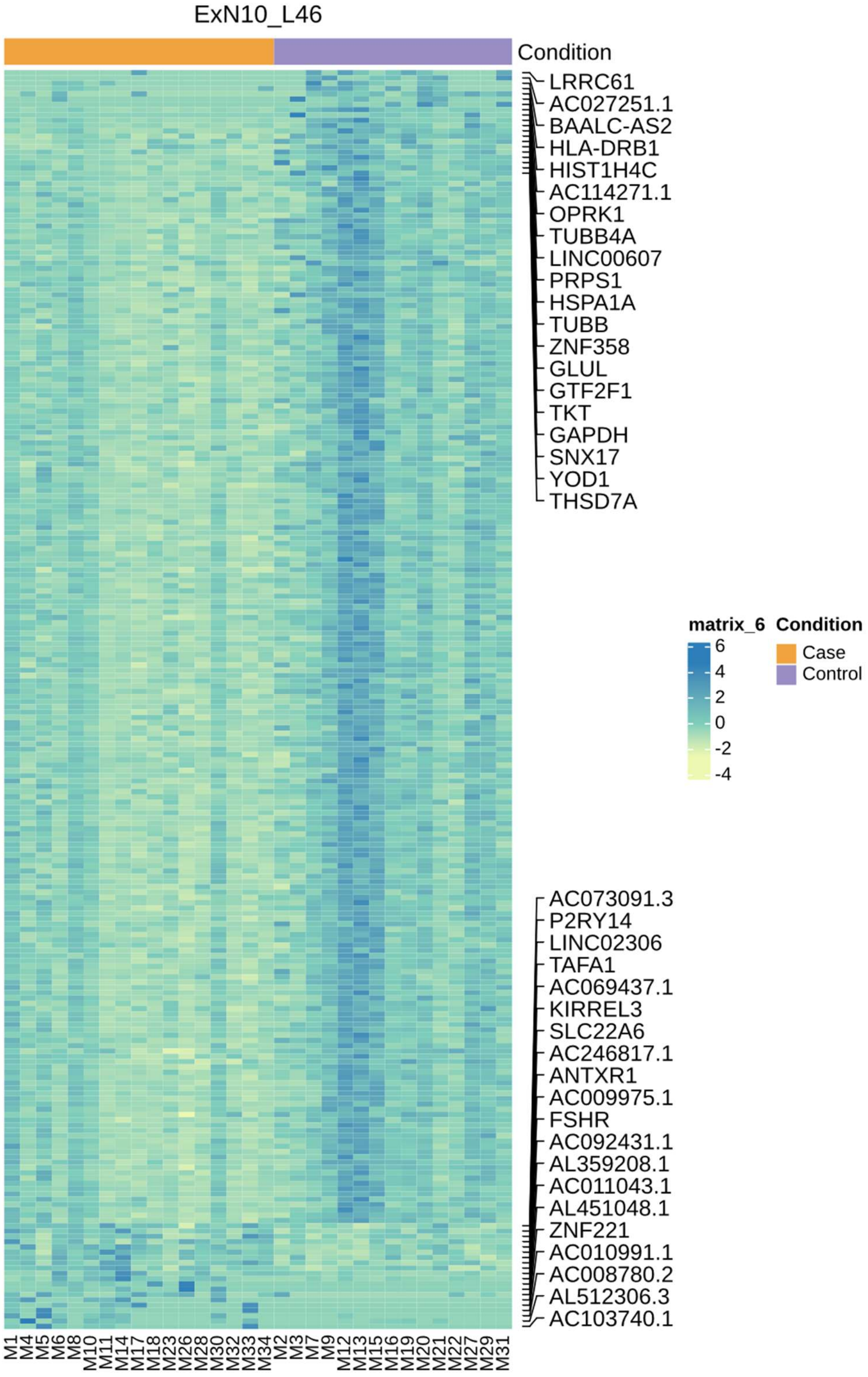
ExN10_L46
Condition
LRRC61
AC027251.1
BAALC-AS2
HLA-DRB1
HIST1H4C
AC114271.1
OPRK1
TUBB4A
LINC00607
PRPS1
HSPA1A
TUBB
ZNF358
GLUL
GTF2F1
TKT
GAPDH
SNX17
YOD1
THSD7A
AC073091.3
P2RY14
LINC02306
TAFA1
AC069437.1
KIRREL3
SLC22A6
AC246817.1
ANTXR1
AC009975.1
FSHR
AC092431.1
AL359208.1
AC011043.1
AL451048.1
ZNF221
AC010991.1
AC008780.2
AL512306.3
AC103740.1
M1 M4 M5 M6 M8 M10 M11 M14 M17 M18 M23 M26 M28 M30 M32 M33 M34 M2 M3 M7 M9 M12 M13 M15 M16 M19 M20 M21 M22 M27 M29 M31
matrix_6
6
4
2
0
-2
-4
Condition
Case
Control

b

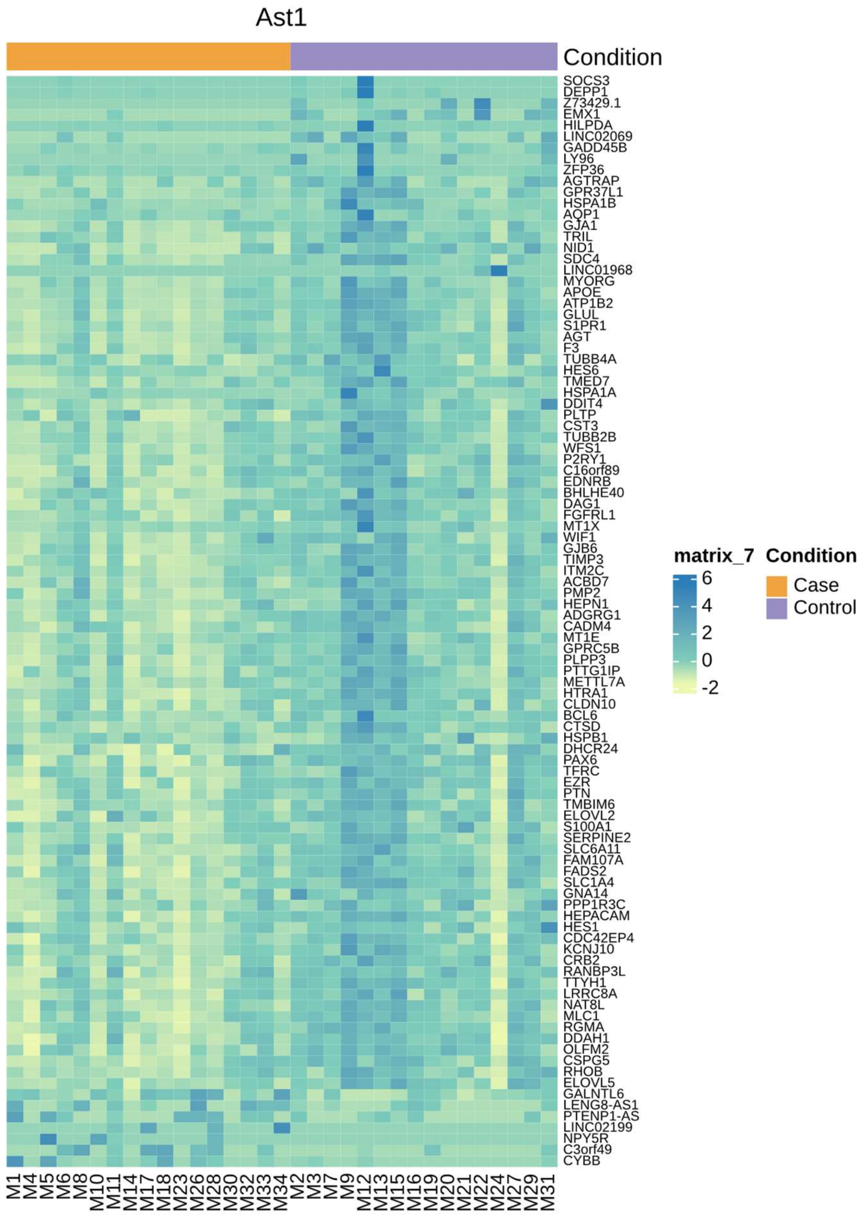

Supplementary Figure 11: DEGs in top clusters for males. a) Heatmaps showing the pseudobulk expression of differentially expressed genes in top clusters with highest number of DEGs in the male cluster level analysis – (a) ExN10_L46, (b) Ast1. The plotted values are pseudobulk CPMs calculated with edgeR and muscat and scaled per row (by gene). The annotation bar at the top is colored orange for cases and purple for controls, and rows and columns are not clustered. For

ExN10_L46 the DEGs are in ascending order of log fold change from top to bottom. Source data are provided as a Source Data file.

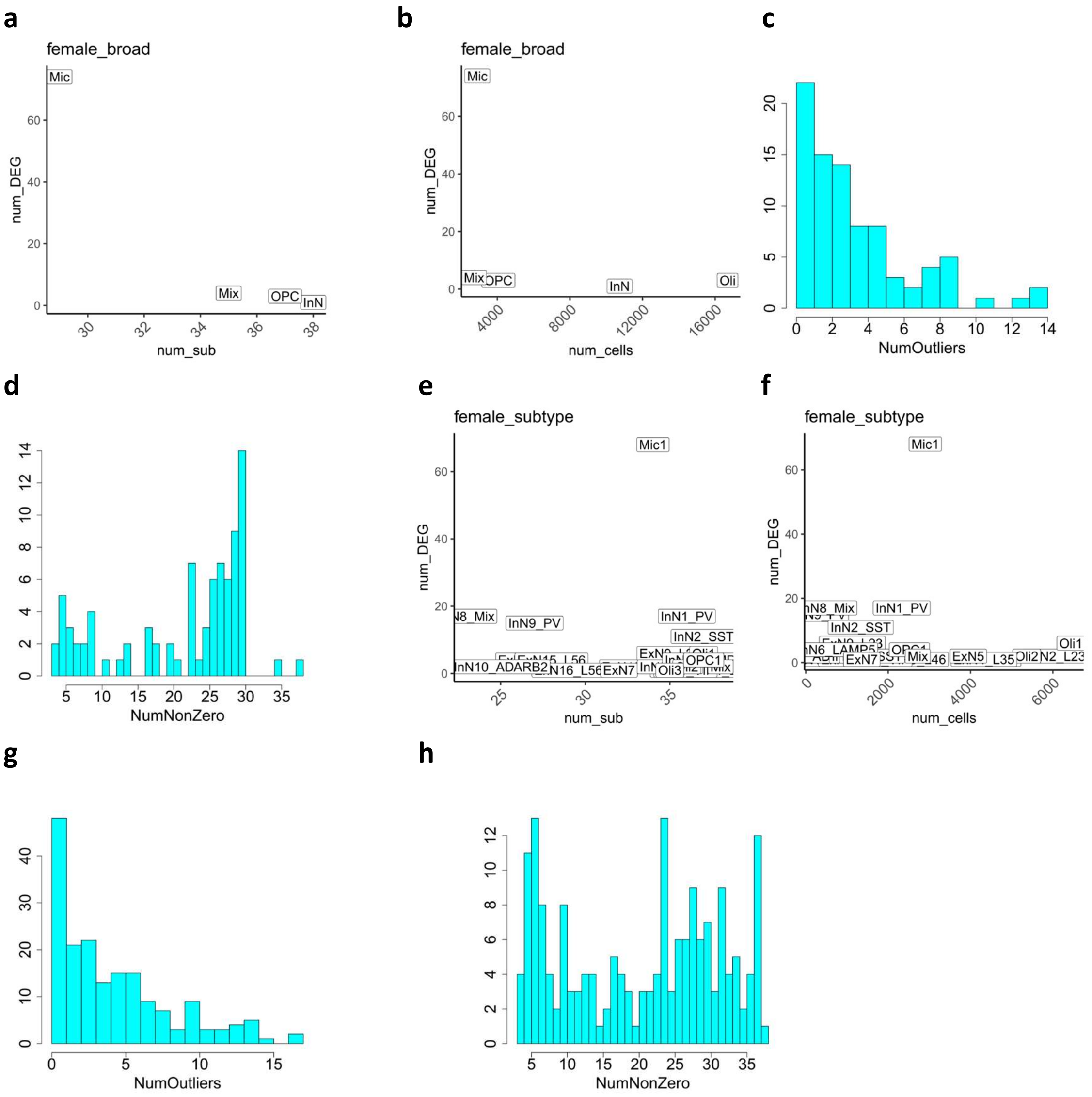

i

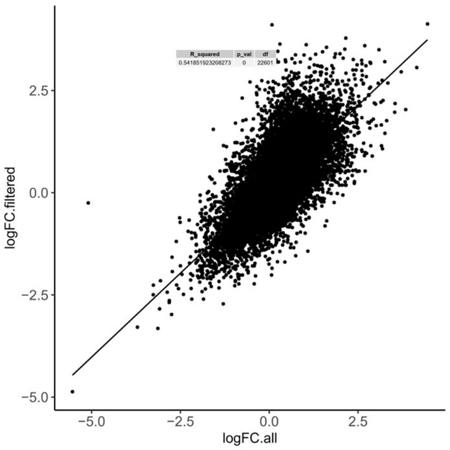

Supplementary Figure 12: Evaluation of female differential expression results. Number of DEGs in each broad cell type for females plotted against (a) the number of subjects included in DEG analysis and (b) the total number of nuclei in the broad cell type. For DEGs in females at the broad cell type level (c) the distribution of number of subjects flagged as possible outliers and (d) the distribution of subjects with non-zero expression. Number of DEGs in each cluster for females plotted against (e) the number of subjects included in DEG analysis and (f) the total number of nuclei in the cluster. For DEGs in females at the cluster level (g) the distribution of number of subjects flagged as possible outliers and (h) the distribution of subjects with non-zero expression. Possible outliers were not removed from the analyses but were assessed as a quality metric for DEG analysis. The plots include the Mix cluster results. i) Scatter plot of log fold changes per gene in DEG analysis of the female microglia data at the cluster level with or without including nuclei that express oligodendroglial markers and cluster close to oligodendroglia on the UMAP plot (methods: Differential expression analysis - Sub-clustering of microglia for differential expression analysis in females). The majority of DEGs obtained with the full microglia cluster were retained in the subsetted microglia cluster (40/68, 59%) and the log fold changes for DEGs were strongly

positively related (linear regression) with an R-squared of 0.54. Source data are provided as a Source Data file.

a

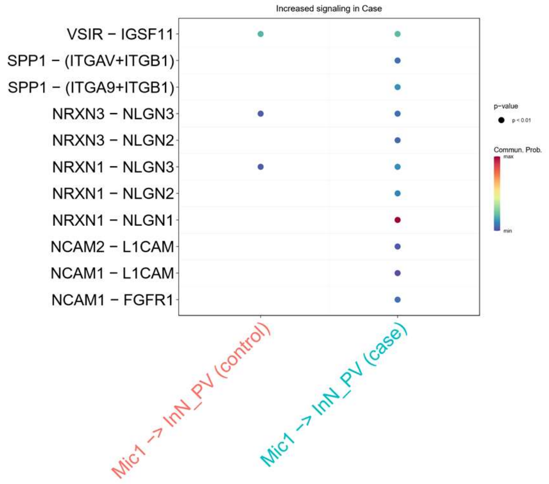

b

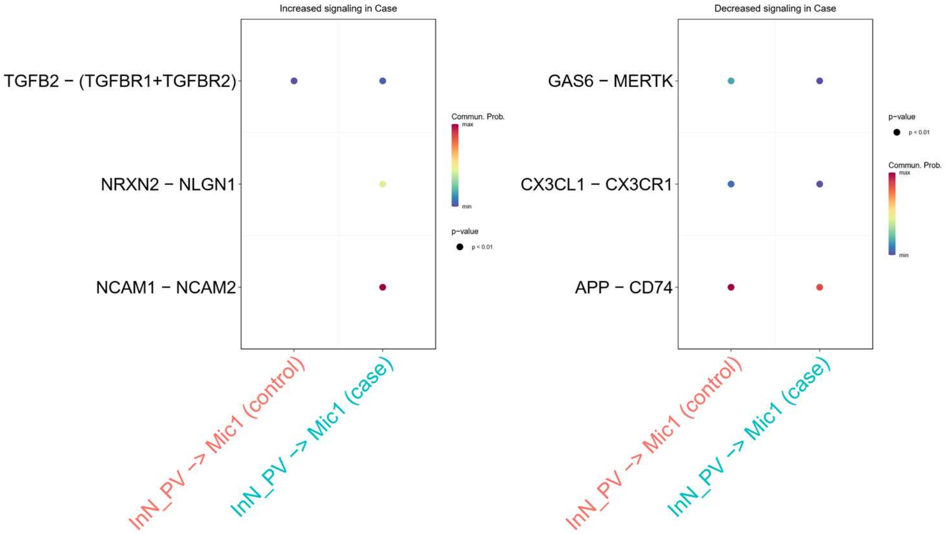

Supplementary Figure 13: Exploratory visualization of ligand-receptor interaction comparison in female microglia and PV interneurons. a) Ligand-receptor pairs with increased signaling from microglia to PV interneurons in cases compared to controls. b) Ligand receptor pairs with increased (left) and decreased (right) signaling from PV interneurons to microglia in cases compared to controls. These results are based on a preliminary assessment using CellChat. The

p-values, corresponding to communication between individual ligand-receptor pairs in cases and controls separately, are based on permutation and are uncorrected. Source data are provided as a Source Data file.

Supplementary References:

1. Maynard, K.R., *et al.* Transcriptome-scale spatial gene expression in the human dorsolateral prefrontal cortex. *Nature neuroscience* 24, 425-436 (2021).
