# Supplementary material for: Cell type specific transcriptomic differences in depression show similar patterns between males and females but implicate distinct cell types and genes
Source: Nat Commun. 2023 May 22;14:2912. doi: 10.1038/s41467-023-38530-5 (PMC10203145; doi:10.1038/s41467-023-38530-5)
Supplement: Supplementary file 2 — Description of Additional Supplementary Files [file 41467_2023_38530_MOESM2_ESM.pdf]

Description of Additional Supplementary Files

File Name: Supplementary Data 1
Description: Sample characteristics and sequencing quality metrics for the male and female cohorts after reanalysis with the Cellranger 5.0.1 pipeline. Column descriptions: Columns G through Y correspond to metrics generated by Cellranger 5.0.1 (https://support.10xgenomics.com/single-cell-gene-expression/software/pipelines/5.0/what-is-cell-ranger) and more information can be found on the 10X Genomics website. Sample - subject and sample code, Condition - MDD case/control status, Batch - Chromium capture batch, Sequencing - sequencing technology, Sex - subject sex, MedianUMI_filtered - median number of UMIs across all nuclei from a sample after nuclei filtering, MedianGene_filtered - median number of genes across all nuclei from a sample after filtering, NumCells_filtered - total number of nuclei from a sample after filtering.

File Name: Supplementary Data 2
Description: Genes enriched in each cluster compared to all other clusters detected using the presto R package "wilcoxauc" function. Column descriptions: feature - gene symbol, group - cluster number, avgExpr - average expression of the gene in the specified cluster, logFC - log fold change of the gene in nuclei from the specified cluster compared to all other nuclei, statistic - statistic returned by the Wilcoxon test, AUC - area under the ROC curve, pval - uncorrected p-value for Wilcoxon test, padj - BH adjusted p-value for the Wilcoxon test, pct_in - percentage of nuclei in the specified cluster expressing the gene, pct_out - percentage of nuclei outisde the specified cluster expressing the gene, group_named - cluster name. Please refer to the presto documentation for further information: https://github.com/immunogenomics/presto. wilcoxauc provides p-values similar to two-sided p-values from the Wilcoxon test.

File Name: Supplementary Data 3
Description: Results from Wilcoxon tests (two.sided) for proportion of nuclei in each broad cell-type or cluster between cases and controls including bootstrapped p-values and p-values after sub-sampling. Column descriptions: For more information on columns C to N refer to the "wilcox_test" function from the rstatix R package. Category - broad cell type or cluster, Name - broad cell type or cluster name, booted_p - p-value produced by bootstrapping the Wilcoxon test, p_adjust - BH adjusted p-value for the Wilcoxon test, booted_p_adjust - BH adjusted booted p-value for the Wilcoxon test, subsampled_p_min, subsampled_p_med, subsampled_p_max - minimum, median, and maximum p-value when running the Wilcoxon test after randomly sub-sampling 70% of nuclei 100 times.

File Name: Supplementary Data 4
Description: Summary of maximal RRHO2 -log10 p-values (hypergeometric tests, one-sided) for threshold free comparison of differential expression results between males and females in broad cell types and clusters. Column descriptions: Name – broad cell type or cluster name, Prominent quadrants with signal – whether the top right and bottom left quadrants (concordant) or the top left and bottom right quadrants (discordant) contain the strongest signal in the RRHO2 plots, Max p val (-log10), Max p val BY corr (-log10) – maximal uncorrected and Benjamini-Yekutieli corrected p-values on a negative log10 scale, Category of evidence – maximal -log10(BY corrected p-value) <15 signifies weak, 15-50 signifies moderate, and >= 50 signifies strong evidence from RRHO analysis, Spearman correlation – Spearman correlation (two-sided) coefficient between male and female datasets using the same gene score as used for RRHO2, Spearman correlation p-value – uncorrected p-value corresponding to the correlation coefficient, Spearman correlation percentile in permutations – fraction of Spearman correlation coefficients obtained using differential expression analysis with permuted case-control labels that are less that the real correlation coefficient. The name is in bold for broad cell types.

File Name: Supplementary Data 5
Description: Differentially expressed genes between cases and controls in males at the broad cell type level and cluster level. Positive logFC indicates increased expression in MDD whereas negative logFC indicates decreased expression in MDD. Column descriptions: For more information on columns A through I please refer to the documentation for the "resDS" function from the muscat R package (https://www.bioconductor.org/packages/release/bioc/html/muscat.html) and the edgeR documention (https://bioconductor.org/packages/release/bioc/html/edgeR.html). NumNonZero - number of subjects with non-zero expression of the gene at the pseudobulk level in the given cluster or broad cell type, NumCaseExcluded, NumControlExcluded - number of MDD cases and number of controls respectively excluded from the analysis as they had too few cells in the specified cluster or broad cell type as described in the methods, Greater3 - whether the gene had non-zero expression in at least 3 subjects, NumOutliers - number of outliers called by the "isOutlier" function from the scater (https://bioconductor.org/packages/release/bioc/html/scater.html) R package, used for quality assessment only. The cluster_id field is in bold for broad cell types. Statistical testing was performed with the edgeR (glmQLFit, glmQLFtest). FDR corrected p-values per cluster are in p_adj.loc.

File Name: Supplementary Data 6
Description: Differentially expressed genes between cases and controls in females at the broad cell type level and cluster level. Positive logFC indicates increased expression in MDD whereas negative logFC indicates decreased expression in MDD. Column descriptions: For more information on columns A through I please refer to the documentation for the "resDS" function from the muscat R package (https://www.bioconductor.org/packages/release/bioc/html/muscat.html) and the edgeR documention (https://bioconductor.org/packages/release/bioc/html/edgeR.html). NumNonZero - number of subjects with non-zero expression of the gene at the pseudobulk level in the given cluster or broad cell type, NumCaseExcluded, NumControlExcluded - number of MDD cases and number of controls respectively excluded from the analysis as they had too few cells in the specified cluster or broad cell type as described in the methods, Greater3 - whether the gene had non-zero expression in at least 3 subjects, NumOutliers - number of outliers called by the "isOutlier" function from the scater (https://bioconductor.org/packages/release/bioc/html/scater.html) R package, used for quality assessment only. The cluster_id field is in bold for broad cell types. Statistical testing was performed with the edgeR (glmQLFit, glmQLFtest). FDR corrected p-values per cluster are in p_adj.loc.

File Name: Supplementary Data 7
Description: Results from Fisher combination of p-values (one-sided) meta-analysis of the differential gene expression results in males and females for broad cell types and cell clusters. Positive logFC indicates increased expression in MDD whereas negative logFC indicates decreased expression in MDD. Only genes with same sign of logFC are listed. Column descriptions: Columns A through I directly correspond to Supplementary Tables 5 and 6 with columns in common having no suffix; columns specific to Supplementary Table 5 having the suffix ".male"; columns specific to Supplementary Table 6 having the suffix ".female". For more information on columns J through L please refer to the documentation for the "fishercomb" function from the metaRNAseq R package (https://cran.r-project.org/web/packages/metaRNASeq/index.html). signs - 1 if both the male and female log fold change for cases versus controls have the same direction and -1 otherwise, type - broad cell type or cluster.

File Name: Supplementary Data 8
Description: re-ranked Gene Set Enrichment Analysis results for clusters with highest numbers of DEGs in females showing only collpased Reactome pathway gene sets. Column descriptions: For more information on columns B through I please refer to the documentation for the "fgseaMultilevel" function from the fgsea (https://bioconductor.org/packages/release/bioc/html/fgsea.html) R package. cluster_id - the cluster for which differential expression results were assessed with fgsea. Statistical testing was performed with preranked gene set enrichment analysis with the adaptive multilevel splitting Monte Carlo approach as implemented in the fgsea package. FDR (Benjamini–Hochberg) corrected p-values are provided.
