# Supplementary material for: Cell type specific transcriptomic differences in depression show similar patterns between males and females but implicate distinct cell types and genes
Source: Nat Commun. 2023 May 22;14:2912. doi: 10.1038/s41467-023-38530-5 (PMC10203145; doi:10.1038/s41467-023-38530-5)
Supplement: Supplementary file 4 — Reporting Summary [file 41467_2023_38530_MOESM4_ESM.pdf]

nature portfolio

Corresponding author(s): Gustavo Turecki

Last updated by author(s): Apr 25, 2023

Reporting Summary

Nature Portfolio wishes to improve the reproducibility of the work that we publish. This form provides structure for consistency and transparency in reporting. For further information on Nature Portfolio policies, see our Editorial Policies and the Editorial Policy Checklist.

Statistics

For all statistical analyses, confirm that the following items are present in the figure legend, table legend, main text, or Methods section.

n/a	Confirmed	
☐	☒	The exact sample size (n) for each experimental group/condition, given as a discrete number and unit of measurement
☒	☐	A statement on whether measurements were taken from distinct samples or whether the same sample was measured repeatedly
☐	☒	The statistical test(s) used AND whether they are one- or two-sided<br>*Only common tests should be described solely by name; describe more complex techniques in the Methods section.*
☐	☒	A description of all covariates tested
☐	☒	A description of any assumptions or corrections, such as tests of normality and adjustment for multiple comparisons
☐	☒	A full description of the statistical parameters including central tendency (e.g. means) or other basic estimates (e.g. regression coefficient) AND variation (e.g. standard deviation) or associated estimates of uncertainty (e.g. confidence intervals)
☐	☒	For null hypothesis testing, the test statistic (e.g. F, t, r) with confidence intervals, effect sizes, degrees of freedom and P value noted<br>*Give P values as exact values whenever suitable.*
☒	☐	For Bayesian analysis, information on the choice of priors and Markov chain Monte Carlo settings
☒	☐	For hierarchical and complex designs, identification of the appropriate level for tests and full reporting of outcomes
☐	☒	Estimates of effect sizes (e.g. Cohen's d, Pearson's r), indicating how they were calculated

Our web collection on statistics for biologists contains articles on many of the points above.

Software and code

Policy information about availability of computer code

Data collection	Cell Ranger v5.0.1 for generating count matrices from FASTQ files
Data analysis	Software used for data analysis: R (v4.0.2, v4.1.2), R packages: Seurat (v4.0.3.9000, v4.0.5), Harmony (v0.1.0), scclusteval (v0.0.0.9000), MetaNeighbor (v1.13.0), slingshot (v2.2.0), tradeSeq (v1.8.0), muscat (v1.8.0), edgeR (v3.36.0), msigdbr (v7.4.1), fgsea (v1.20.0), psygenet2r (v1.26.0), CellChat (v1.4.0), metaRNASeq (v1.0.7), RRHO2 (v1.0), tidyverse (1.3.1), QuPath (0.2.0).<br>No custom software was developed for data analysis, but the scripts used to apply the published software to our data are provided here: https://github.com/MgssGroup/snRNASeq_public/.

For manuscripts utilizing custom algorithms or software that are central to the research but not yet described in published literature, software must be made available to editors and reviewers. We strongly encourage code deposition in a community repository (e.g. GitHub). See the Nature Portfolio guidelines for submitting code & software for further information.

Data

Policy information about availability of data

All manuscripts must include a data availability statement. This statement should provide the following information, where applicable:

- Accession codes, unique identifiers, or web links for publicly available datasets
- A description of any restrictions on data availability
- For clinical datasets or third party data, please ensure that the statement adheres to our policy

Raw sequencing data (FASTQ files) for the female cohort generated in this study is available on GEO (accession number: GSE213982) along with the processed gene-barcode matrix and metadata including both male and female cohorts. The raw sequencing data for the male cohort are also available on GEO (accession number: GSE144136). We used STRING-DB (v11.5) for protein-protein interaction analysis. Additionally, the processed data from this study has been uploaded to the UCSC Cell Browser for easy visualization: https://dlpfc-mdd.cells.ucsc.edu. Source data for all figures in this paper are provided on Zenodo: https://doi.org/10.5281/zenodo.7884086. The reference genome version used is available on the 10X Genomics website (refdata-gex-GRCh38-2020-A, https://support.10xgenomics.com/single-cell-gene-expression/software/release-notes/build).

Allen Brain Institute motor cortex data used for MetaNeighbor comparison are available for download here: https://portal.brain-map.org/atlases-and-data/rnaseq/human-m1-10x. STAB reprocessed data from published snRNA-seq and scRNA-seq datasets used for MetaNeighbor comparison is available here: https://mai.fudan.edu.cn/stab/help/. The spatial transcriptomics data used here for label transfer is available using the spatialLIBD (version 1.6.0) R package and through the AWS download links provided here: https://github.com/LieberInstitute/spatialLIBD.

Human research participants

Policy information about studies involving human research participants and Sex and Gender in Research.

Reporting on sex and gender	We considered sex as a factor of interest in our analysis. Sex was assigned to postmortem samples based on medical records and information from next-of-kin. Our current study focuses on female subjects with and without MDD. We further combined our findings in females and our findings in males in a meta-analysis. We do not have enough information from the post-mortem samples to accurately assess gender at this time.
Population characteristics	Population characteristics: Human post-mortem female brains: 20 MDD cases (age 45.10±3.19 years) and 18 neurotypical controls (47.89±4.45 years). Human post-mortem male brains: 17 MDD cases (41.06±4.66 years), 16 neurotypical controls (38.38±4.58 years)<br><br>One female case subject and three female control subjects were Hispanic, two female control subjects were African American, and race information was missing for one female case. All other female subjects were Caucasian, as were all subjects in the male cohort.<br><br>Values for age represent mean and SEM.
Recruitment	Samples were obtained from the Douglas Bell Canada Brain Bank in collaboration with the Quebec coroner's office and the University of Miami Miller School of Medicine Brain Endowment Bank.
Ethics oversight	This study was approved by the Douglas Institute IRB.

Note that full information on the approval of the study protocol must also be provided in the manuscript.

Field-specific reporting

Please select the one below that is the best fit for your research. If you are not sure, read the appropriate sections before making your selection.

☒ Life sciences ☐ Behavioural & social sciences ☐ Ecological, evolutionary & environmental sciences

For a reference copy of the document with all sections, see nature.com/documents/nr-reporting-summary-flat.pdf

Life sciences study design

All studies must disclose on these points even when the disclosure is negative.

Sample size	We did not perform sample-size calculation. Sample size of 20 MDD cases and 18 neurotypical controls was chosen based on recent snRNA-seq studies of post-mortem human brain tissue in the context of neuropsychiatric conditions, which had similar numbers of samples. The number of subjects included in our study is comparable to published snRNA-seq studies of neuropsychiatric conditions, which have included between 11 to 48 subjects (Lau et al., 2020 – PMID 32989152; Mathys et al., 2019 – PMID 31042697; Morabito et al., 2021 – PMID 34239132; Smajić et al., 2022 – PMID 34919646; Velmeshev et al., 2019 – PMID 31097668).
Data exclusions	We filtered low quality nuclei based on numbers of UMIs, numbers of genes, and percentage of mitochondrial genes. We removed any samples from differential expression analysis if they had less than 5 nuclei in the given cluster or less than 10 nuclei in the broad cell type. The

same exclusion criteria was applied to the WGCNA, as used for differential analysis. Data exclusion criteria are described in detail in the methods.

Replication

We did not attempt to replicate our results in the newly generated female cohort. We did not attempt to replicate our findings in males in an independent cohort, however, our results in the male cohort are similar to what we reported in a previous analysis of the same cohort. Large scale studies of postmortem brain tissue are logistically challenging as recruiting sufficient numbers of well characterized subjects can take many years. Thus an independent validation could not be performed as part of this study.

Randomization

We did not randomize samples into groups as we are studying postmortem human brain from individuals without or without MDD. Case samples were selected from the pool of recruited brains in the Douglas Bell Canada Brain Bank by an administrator based on positive diagnosis of depression, as described in the methods. We selected control subjects with comparable pH, PMI, and age as much as possible. We corrected for age, pH, PMI, and batch in our differential expression analysis and our WGCNA.

Blinding

Clinicians were blinded for final psychiatric autopsy diagnosis of MDD case or control. Blinding was not performed during data generation since the case and control information was used to balance representation of both groups in each processing batch. Clustering and annotation of nuclei was performed in an unbiased blinded manner. Blinding was not performed during differential expression analysis or WGCNA since the case and control annotations are required for performing these analyses.

Reporting for specific materials, systems and methods

We require information from authors about some types of materials, experimental systems and methods used in many studies. Here, indicate whether each material, system or method listed is relevant to your study. If you are not sure if a list item applies to your research, read the appropriate section before selecting a response.

Materials & experimental systems

n/a	Involved in the study
☒	☐ Antibodies
☒	☐ Eukaryotic cell lines
☒	☐ Palaeontology and archaeology
☒	☐ Animals and other organisms
☒	☐ Clinical data
☒	☐ Dual use research of concern

Methods

n/a	Involved in the study
☒	☐ ChIP-seq
☒	☐ Flow cytometry
☒	☐ MRI-based neuroimaging
